# Serum cystatin C is a determinant of central pressure augmentation index measured by oscillometric method in renal transplant recipients

**DOI:** 10.1186/1471-2369-15-196

**Published:** 2014-12-11

**Authors:** Miriana Dinic, Nicolas Maillard, Damien Thibaudin, Martin Jannot, Ingrid Masson, Eric Alamartine, Christophe Mariat

**Affiliations:** Service de Néphrologie, Transplantation Rénale, Dialyse, Centre d’Hypertension Artérielle, Hôpital Nord, CHU de Saint-Etienne, Université Jean Monnet, PRES Université de Lyon, 42055 Saint Etienne, Cedex 04, France

**Keywords:** Cystatin C, Arterial stiffness, Cardiovascular risk, Renal transplantation

## Abstract

**Background:**

Serum cystatin C (ScysC) may help predicting cardiovascular outcome not only through its ability to detect renal dysfunction but also through its potential connection to others factors that are directly related to cardiovascular diseases. We explored the potential association of ScysC with arterial stiffness - a major contributor to cardiovascular disease - in renal transplant recipients (RTR).

**Methods:**

Traditional and non-traditional cardio-vascular risk factors were collected from 215 stable RTR whom arterial stiffness was evaluated by the measure of the augmentation index of central pressure (AIx) determined by the arteriograph device. Serum creatinine and ScysC were measured the same day using standardized methods. Association between ScysC and AIx was examined in univariate and multivariate linear regression analysis.

**Results:**

In univariate analysis, ScysC was strongly associated with AIx. This relationship was not confounded by age, gender, length of time spent on dialysis and transplantation vintage. Adjustment on the level of GFR estimated by the MDRD Study equation attenuated but did not abolish the association between ScysC and AIx.

**Conclusions:**

In conclusion, ScysC is an independent predictor of AIx in RTR. Our data suggest that arterial stiffness may partially mediate the association between ScysC and cardiovascular risk in renal transplantation.

## Background

Cardiovascular diseases are particularly prevalent in renal transplant recipients (RTR) and represent a leading cause of patients’ death and graft loss [1, 2]. Besides traditional risk factors such as dyslipidemia, diabetes or hypertension, others factors more specific to RTR are thought to be in play and to explain the excess of risk seen in transplantation. Those non-traditional factors (e.g. calcineurin inhibitor use, length of time spent on dialysis) are not taken into account by the commonly used risk-prediction equations, which tend to systematically underestimate the risk of RTR [3, 4]. More importantly, identification and characterization of unconventional factors that might significantly improve risk prediction in RTR is still limited.

The interest of Serum cystatin C (ScysC) as an alternative –and somehow superior - GFR marker to serum creatinine has recently been highlighted in the general population and in patients with native chronic kidney disease (CKD) [5]. Beyond its role in evaluating renal function, ScysC is more and more regarded as a potential cardiovascular risk factor [6, 7]. A close relationship between ScysC and cardiovascular morbi-mortality has been reported in the general population as well as in patients with various degrees of CKD [8]. In renal transplantation, we have confirmed the validity of ScysC as an endogenous marker of renal graft function [9] and have recently reported a stronger association (as compared to serum creatinine) between ScysC concentration and RTR mortality [10].

Interestingly, the ability of ScysC to predict cardiovascular outcome is thought to be explained not only by the ability of ScysC to detect renal function impairment (which is by itself a strong cardiovascular risk factor) but also by its possible connection to others factors that are directly related to cardiovascular diseases [11]. In this regard, ScysC has recently been suggested, in the general population [12–15], to be independently associated with excessive arterial stiffness, a condition that has attracted considerable interest in cardiovascular epidemiology [15].

Arterial stiffness is currently considered by many as a major contributor - independent to classical cardio-vascular risk factors- for cardiovascular mortality, coronary events and strokes in the general population [16, 17] and in patients with essential hypertension [18, 19], type 2 diabetes [20], or end-stage renal disease [21, 22]. From a mechanistic standpoint, excessive arterial stiffness is responsible for a premature return of the reflected pulse wave in the late phase of systole, which causes an increase in central pulse pressure and thus an excess of workload and oxygen demand for the left ventricle. The augmentation of central pulse pressure may also directly influence arterial remodeling and accelerates the development of stenosis and plaques in cerebral arteries. In renal transplantation, arterial stiffness has been associated with the occurrence of cardiovascular events and an excess of mortality suggesting that it also acts as a significant contributor of cardio-vascular diseases in this particular setting [23].

Herein, we aimed to better understand the cardiovascular dimension of ScysC in renal transplantation. We sought to verify whether the association between ScysC and arterial stiffness seen in the general population could extend to RTR and explored the confounding role of major traditional and non-traditional transplant-related cardiovascular risk factors.

## Methods

### Patients

From December 2012 to August 2013, all prevalent renal transplant recipients with a functioning graft and attending the outpatient clinics at the Saint Etienne University Hospital for their annual check-up were eligible for the study. Inclusion criteria were age ≥18 years, time after transplantation ≥6 months and a stable clinical condition. This study was approved by the institutional review board (Comité de Protection des Patients, reference number 2012–35) and written informed consent obtained from each participant. The investigation conformed to the principles outlined in the Declaration of Helsinki. This study is non-controlled, cross-sectional evaluation of central hemodynamics parameters in renal transplant patients.

As part of the standard check-up, the following clinical parameters were collected: weight, height, abdominal perimeter, brachial artery blood pressure in triplicate using a validated oscillometric technique (HEM_7223 Omron Healthcare, Kyoto, Japan). We systematically interrogated patients and inventoried patients’ file for personal and family history of cardiovascular disease (defined as a first-degree male relative having suffered a cardiovascular disease before the age of 55, or a first-degree female relative having suffered one before the age of 65), diabetic and smoking status, anti-hypertensive drugs (number and type), immunosuppressive drugs (type and posology), history of acute or chronic graft rejection, age at the time of renal transplant, total time on renal replacement therapy and type of renal replacement therapy, etiology of native nephropathy, characteristics of the donor (age, height), cold ischemia time. The Kauppila abdominal aortic calcification score [24] was assessed from a lateral lumbar X-ray realized the day of the annual visit.

### Laboratory measurements

All biological parameters were measured the same day and at a single laboratory. Serum creatinine and ScysC were both measured using standardized methods, IDMS-traceable enzymatic method (Crea Vitros) and IFCC-traceable N Latex cystatin kit (Siemens), respectively. The following parameters were additionally measured: calcemia, phosphoremia, magnesemia, total cholesterol blood level, high and low-density lipoprotein cholesterol, hematocrit, hemoglobin, HbA1C, 25 hydroxy-vitamin D, parathyroid hormone, proteinuria, micro-albuminuria, C-reactive protein, immunosuppressive drug trough levels (tacrolimus, cyclosporine, everolimus and sirolimus), peripheral CD4+ cells count, cytomegalovirus PCR, and homocysteinemia.

### Assessment of arterial stiffness

**Figure 1:**
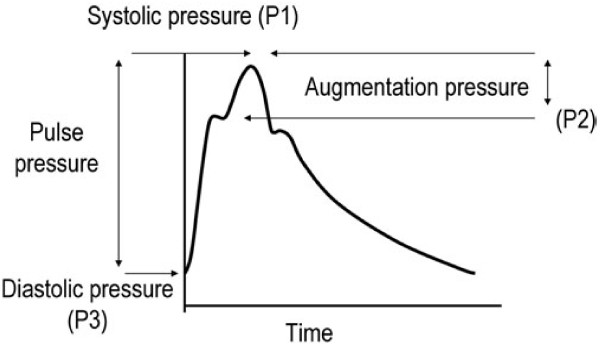
**Pulse wave analysis.** The arterial pressure waveform is the sum of the forward wave following ventricular contraction and a backward wave reflected from the periphery. In vessels with normal elasticity, the wave velocity is sufficiently low to allow reflection of the backward wave at the aortic root during diastole. In the case of increased stiffness, velocity increases causing a premature reflection of the backward wave during systole. This phenomenon is quantified through the augmentation index (AIx)—defined as the difference between the second and first systolic peaks (P1-P2, absolute augmentation pressure) expressed as a percentage of the pulse pressure (P1-P3).

Central Hemodynamic Parameters (central blood pressure, central pressure augmentation index and pulse wave velocity) were recorded in the non-fistula arm using a validated oscillometric device (Arteriograph®, Medexpert). Only the central pressure augmentation index (AIx) was considered for the analysis of arterial stiffness in this study (Figure 1).

### Statistical analysis

Descriptive statistics were used to evaluate recipient-, donor- and transplantation-related characteristics. Quantitative data are expressed as means ± SD and as percentage for categorical variables.

Linear regression analysis was performed to assess the association between different co-variables and AIx. A first selection of potentially interesting variables was made based on their theoretical and/or described impact on arterial stiffness. The relationship between each of these variables and AIx was first tested in univariate models. The coefficients associated to each variable were displayed with their 95% confidence interval as well as their p value from t test. Intercepts were not displayed. Multivariate models were then built in a forward manner to optimize the overall goodness-of-fit of the model, by maximizing R^2^ adjusted on the number of covariates. Variables that were the most strongly associated to AIx (p < 0.05 in univariate models) were eligible to be integrated to the multivariate models and only those which resulted in adjusted R^2^ improvement were finally retained. A p-value associated to a given coefficient below 0.05 and a 95% confidence interval outside of the 0 value were considered to be significant. Each model was checked to provide normally distributed residuals (quantile-quantile plots, not displayed).

Model comparison was performed with ANalysis Of VAriance (ANOVA). The p-value associated to F-statistics below 0.05 was considered as a significant improvement of the accuracy of the second tested model over the first one.

A bivariate cubic spline regression model was built to predict AIx from both creatinine and Cystatin C. Residuals from this model were normally distributed. This model was illustrated in a 3-dimensional chart.

Data were analysed using R software (Version 2.15.2). R core team (2012). R : A language and environment for statistical computing, Vienna, Austria. ISBN 3-900051-07-0, http://www.R-project.org/.

## Results

### Demography

Two hundred and fifteen RTR were retained for the analysis. Characteristics of the population at the time of assessment are shown in Table 1. Importantly, RTR were almost exclusively transplanted from a deceased donor (4.2% of living donor), were treated for hypertension for more than 85% of them and were receiving a calcineurin inhibitor-based regimen for more than 95% of them. Mean serum creatinine and serum cystatin C were 140.7 μmol/l and 1.68 mg/l, respectively. Mean GFR estimated by the MDRD Study equation was 52.2 ml/min/1.73 m^2^. Descriptive statistics of the central hemodynamic parameters are shown in Table 2.

**Table 1:** **Demography**

Parameters	All patients (n = 215)
Age (years)	55.9 ± 13
Time since transplantation (years)	9.2 (0.5-34)
Time spent on dialysis (years)	3 (0–26.3)
Gender (male) (%)	67.4
Type of dialysis (HD) (%)	81.4
Height (m)	1.68 ± 0.07
BMI (kg/m^2^)	25 ± 5.8
Abdominal perimeter (cm)	93.1 ± 13.1
Current smoker (%)	14.9
Diabetes (%)	18.6
Personal cardiovascular history (%)	16,3
Coronaropathy (%)	10,2
Stroke (%)	6
Peripheral arteriopathy (%)	3,3
Serum Creatinine (μmol/l)	140.7 ± 55.3
eGFR (ml/min/1.73 m^2^)	52 ± 22
Serum Cystatin C (mg/l)	1.68 ± 0.65
Serum Homocystein (μmol/l)	18.6 ± 8.2
CRP (mg/l)	9,2 ± 20.6
Glycemia (mmol/l)	6.2 ± 8.3
Cholesterol total (mmol/l)	4.9 ± 1.1
HDL-cholesterol (mmol/l)	1.35 ± 0.42
Calcemia (mmol/l)	2.69 ± 0.68
Phosphoremia (mmol/l)	1.16 ± 0.16
Magnesemia (mmol/l)	0.70 ± 0.08
Parathormone (ng/l)	129.68 ± 60.84
HR (bpm)	67 ± 9.7
Brachial SBP (mm Hg)	138 ± 17.1
Brachial DBP (mm Hg)	77.7 ± 10.2
Aortic calcification (mean Kauppila score)	2.8 (0–24)
Treatment	
ACE inhibitors/ARB (%)	63,3
Beta-blockers (%)	38,1
Calcium channel blockers (%)	49,8
Diuretics (%)	40,9
Aziathropine (%)	8,8
MMF (%)	60,5
Cyclosporine (%)	12,1
Cyclosporine level (μg/l)	81,7 ± 51,3
Tacrolimus (%)	84,7
Tacrolimus level (ng/ml)	6,4 ± 2,1
mTOR inhibitor	8,4
mTOR level (ng/ml)	4,4 ± 2
Corticosteroids (%)	15,3

*HR*, Heart Rate; *SBP*, Systolic Blood pressure; *DBP*, Diastolic Blood Pressure; *ACE*, Angiotensin Converting Enzyme; *ARB*, Angiotensin Receptor Blockers.

**Table 2:** **Central hemodynamic parameters**

	Mean ± SD	Median	Range
Aortic SBP (mm Hg)	131.1 ± 23.1	126.3	88.7 - 224.7
Aortic pulse pressure (mm Hg)	47.8 ± 16	44	25.2 - 113.7
Aortic AIx (75)	25.7 ± 15.3	24.9	-3.2 - 59.1
Aortic PWV (m/s)	9.3 ± 1.8	9.3	4.9 - 15.2

*SBP*, Systolic Blood Pressure; *PWV*, Pulse Wave Velocity.

### Association between ScysC and AIx

**Figure 2:**
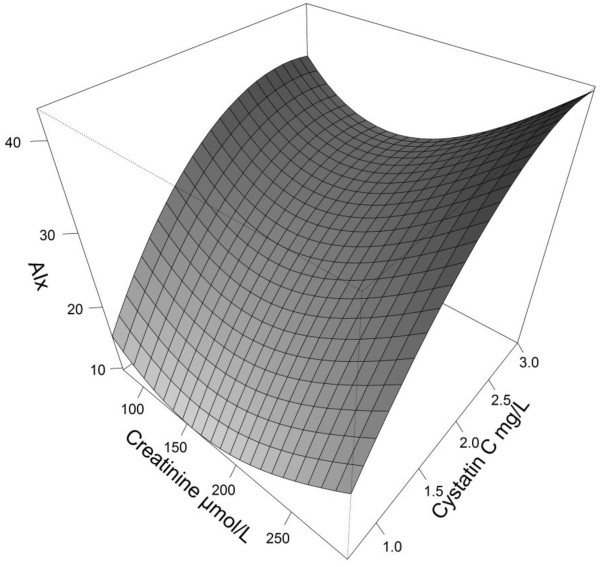
**Three dimensional representation of a bivariate spline regression model to predict AIx from both Creatinine and Cystatin C.**

ScysC was positively associated with AIx following a monotonic increasing curve. In comparison, the relation between serum creatinine and AIx followed a J-shaped curve (Figure 2). In univariate analysis, ScysC was significantly associated with AIx (P <0.001). All significant covariates are displayed in Table 3. In contrast, the following variables turned out to be not significantly associated with AIx: albuminuria, diabetes and smoking status, LDL and HDL cholesterol, C reactive protein, vitamin D, familial history of cardiovascular diseases, renin-angiotensin system blockers, calcineurin use and systemic exposure, use of steroids, history of chronic rejection, CD4+ lymphopenia, CMV replication.

**Table 3:** **Univariate predictors of Aortic AIx (75)**

Variable	Estimate	Confidence interval 95%	p value
Age (per year)	0.40	0.25 to 0.54	<0.001
Gender (Female vs. Male)	7.12	2.87 to 11.4	0.001
Height (per meter)	-35.4	-58.2 to -12.6	0.003
Transplantation time (per year)	-0.348	-0.65 to -0.050	0.022
Time on dialysis (per year)	0.730	0.16 to 1.29	0.012
Dialysis method (Peritoneal dialysis and none vs. Hemodialysis)	5.76	0.54 to 11.0	0.03
Personal Cardiovascular history (yes vs. no)	6.98	1.49 to 12.5	0.013
SBP (per mmHg)	0.34	0.24 to 0.43	<0.001
DBP (per mmHg)	0.33	0.18 to 0.48	<0.001
Diuretic use (yes vs. no)	6.86	2.49 to 10.7	0.002
eGFR (per mL/min/1.73 m2)	-0.187	-0.28 to -0.10	<0.001
Cystatin C (per mg/L)	7.69	4.43 to 10.9	<0.001
Mg (per mmol/L)	26.2	6.6 to 45.7	0.008
Homocysteinemia (per mg/L)	0.40	0.15 to 0.65	0.002
PTH (per ng/L)	0.030	0.006 to 0.054	0.014
Ca (per mmol/L)	-19.82	-32.8 to -6.77	0.003
P (per mmol/L)	16.2	7.2 to 25.1	<0.001
Kauppila score (per unit)	0.94	0.49 to 1.39	<0.001

Estimates represent the variation of AIx for each unit of the covariate. P values below 0.05 mean that the covariate is significantly associated to AIx.

*SBP*, Systolic Blood Pressure; *DBP*, Diastolic Blood Pressure; *eGFR*, Estimated Glomerular Filtration Rate (MDRD Study Equation); *Mg*, Magnesiemia; *PTH*, Parathyroid Hormone; *Ca*, Calcemia; *P*, Phosphatemia.

### Multivariate analysis

Only variables significantly associated with AIx in the univariate analysis and that resulted in an increase of the model adjusted R^2^ were retained in the final multivariate regression analysis. Different models with increasing performance were successively built. Model 1 included age, time on dialysis, transplantation time and SBP. Integration of ScysC into this model substantially improved its overall performance (Model 2). After adjustment for eGFR (Model 3) and for height and gender (Model 4), the association between AIx and ScysC was attenuated but remained statistically significant (Table 4).

**Table 4:** **Multivariate linear regression models**

	Estimate	Confidence interval 95%	p value	R ^2^
***Model 1***			<0.001	0.302
Intercept	-27.1	-42.1 to -12.1	<0.001	
Age	0.319	0.163 to 0.475	<0.001	
Time on dialysis	0.781	0.289 to 1.273	0.002	
Transplantation time	-0.403	-0.682 to -0.124	0.004	
SBP	0.275	0.174 to 0.375	<0.001	
***Model 2***			<0.001	0.345
Intercept	-28.8	-45.0 to -12.6	<0.001	
Age	0.251	0.07 to 0.428	0.006	
Time on dialysis	0.745	0.231 to 1.258	0.005	
Transplantation time	-0.456	-0.764 to -0.148	0.004	
SBP	0.245	0.136 to 0.355	<0.001	
**Cystatin C**	5.81	2.60 to 9.03	<0.001	
***Model 3***			<0.001	0.346
Intercept	-24.5	-46.7 to -2.34	0.03	
Age	0.246	0.067 to 0.425	0.007	
Time on dialysis	0.741	0.223 to 1.26	0.005	
Transplantation time	-0.453	-0.764 to -0.141	0.005	
SBP	0.245	0.133 to 0.357	<0.001	
Cystatin C	4.82	0.234 to 9.40	0.039	
**eGFR**	-0.045	-0.185 to 0.095	0.53	
***Model 4***			<0.001	0.388
Intercept	3.23	-60.6 to 67.0	0.92	
Age	0.241	0.066 to 0.416	0.005	
Time on dialysis	0.636	0.123 to 1.15	0.01	
Transplantation time	-0.436	-0.741 to -0.130	0.005	
SBP	0.266	0.155 to 0.376	<0.001	
Cystatin C	5.70	1.19 to 10.20	0.01	
eGFR	-0.012	-0.151 to 0.127	0.87	
**Height**	-22.3	-53.9 to 9.17	0.16	
**Gender**	3.27	-2.89 to 9.43	0.3	

P values below 0.05 mean that the covariate is significantly and independently associated to AIx.

*SBP*: Systolic Blood Pressure. *eGFR*: Estimated-GFR estimation (MDRD Study equation). Variables displayed with a bold font are added to the previous model.

In order to better apprehend the added value of ScysC beyond its capacity to reflect GFR, we compared the performance of models based on eGFR and integrating or not ScysC. Interestingly, adding ScysC in both univariate and multivariate eGFR-based models significantly improved the overall performance of the model (Table 5).

**Table 5:** **Comparison of eGFR based models before and after adding serum Cystatin C as a covariable (ANOVA)**

	R ^2^ without Cystatin C	R ^2^ with Cystatin C	F statistics	p value
eGFR alone vs. (eGFR + Cystatine C)	0.090	0.114	4.14	0.044
Model 4 vs. (Model 4 – Cystatin C)	0.362	0.388	6.25	0.014

Addition of Cystatin C significantly improved both univariate and multivariate mGFR based models.

## Discussion

Cardiovascular disease is an important cause of morbidity and mortality in patients with chronic kidney disease. This is thought to be explained by a phenomenon of premature aging of the vascular system which results in an increase of arterial stiffness in this population. The pathophysiology of this vascular disorder is not well understood. Importantly, while arterial stiffness has been well characterized in pre-dialysis and dialysis patients, few data are available for transplant patients [25].

The main finding of our study is that ScysC is significantly associated to AIx in RTR and so, independently of other covariates known to be associated with AIx and/or to cardiovascular morbi-mortality. In this respect, we sought to incorporate in our analysis not only the traditional confounders (such as age, systolic blood pressure, diabetes, dyslipidemia) but also more transplant-specific factors that have been previously connected to AIx (donor age [26], magnesemia [27] or cardiovascular complications (aortic calcification [24], CMV replication [28], CD4+ lymphopenia [29]). In addition, the association between ScysC and AIx remained independent after adjustment to eGFR suggesting that an increase in ScysC concentration might not only reflect impairment in renal function but also to a certain extent excessive arterial stiffness. The partial independency of ScysC from renal function would however need to be confirmed by analyzing “true” GFR (i.e. a GFR measured using a reference method) rather than an estimate of GFR. Obviously, the alternative explanation is that ScysC, simply by reflecting more accurately renal function than serum creatinine, is more strongly associated to AIx. This latter hypothesis is in line with previous data showing that (i) renal function contributes to arterial stiffness [30], and (ii) that ScysC is a better GFR maker than serum creatinine in renal transplantation [9]. Regardless of the underlying mechanism and given that the current guidelines recommend to evaluating the level of renal function and its related risk with creatinine-based estimates [31], we believe that the eGFR-independent connection of ScysC to AIx is, from a practical point of view, relevant.

To our knowledge, our study is the first to report an association between ScysC and AIx in renal transplantation. Previous studies have however reported similar association between ScysC and arterial stiffness in older adults [13] and in the general population with no apparent CKD [12, 14]. At odds with those studies, ours did not rely on the direct measure of pulse wave velocity (PWV) by applanation tonometry (the real gold standard of arterial stiffness evaluation) but on the measure of central pressure augmentation (i.e. a proxy of PWV) using a more convenient and less operator-dependent oscillometric method. Importantly, this method has been validated against the gold standard approach and has already been utilized in a study reporting an association between ScysC and AIx in hypertensive patients [15].

Irrespective of the abiblty of ScysC to account for renal dysfunction, the biological properties of ScysC can provide some physiopathological basis to its association with AIx. Cystatin C is a cysteine protease inhibitor that controls the activity of cathepsins. Through their elastolytic and collagenolytic activities, cathepsins may contribute to atherosclerogenesis. Overexpression and overactivity of cathepsins are found in human atherosclerotic lesions [32]. In response to this overexpression, cystatin C production might be stimulated to regulate cathepsins activity. In this purely speculative scenario, elevated ScysC concentration would only be an indirect marker of cathepsin-induced arterial stiffness.

Several strengths of our study can be pointed. First, this is a single-center, prospectively planned study with careful selection of all clinical variables and centralized measurement of all biological variables. Measurement of serum creatinine and ScysC were both standardized. Second, we selected co-variates of interest not only based on the literature available in the general population but also with a special focus on risk factors specifically identified in previous renal transplant cohorts. Third, we sought to analyze certain covariates in the most informative way possible: the “immunosuppressive drugs” variable was assessed according to the type of drug and to the systemic exposure to the drug; the “anti-hypertensive treatment” covariate, according to the type and to the number of drugs.

Several limitations of our study must similarly be considered. First of all, the cross-sectional design of our study precludes us from making any causal inference in the association between covariates and AIx. Even the direction of the association might be difficult to ascertain in this context. Since our patients are exclusively Caucasians, transplanted mainly form deceased donors, our results may not be generalizable to other populations. Additionally, we cannot rule out the possibility of unmeasured confounding from variables that were not evaluated in our study (e.g. we did not include in our analysis the presence of a functioning fistula, a variable that has been previously associated with arterial stiffness in RTR [33]). Finally, since GFR was not directly measured by a reference method, we cannot clarify whether the association between ScysC and AIx is or is not solely explained by the association between ScysC and renal function.

## Conclusions

In conclusion, elevated ScysC concentration is independently associated with AIx in stable RTR. This relationship is not confounded by age, gender, length of time spent on dialysis and transplantation vintage neither by the level of GFR estimated by the MDRD Study equation. Our data suggest that arterial stiffness may partially mediate the association between ScysC and cardiovascular risk in renal transplantation. Whether the association between ScysC and arterial stiffness reflects the direct implication of ScysC in atherosclerogenesis or solely its ability to approximate GFR will need further research.

## Abbreviations

AIx: Augmentation index
PWV: Pulse wave velocity
PP: Pulse pressure
RTR: Renal transplant recipients
BMI: Body mass index
HD: Hemodialysis
eGFR: Estimated glomerular filtration rate
SBP: Systolic blood pressure
DBP: Diastolic blood pressure
HR: Heart rate
ACE: Angiotensin converting enzyme
ARB: Angiotensin receptor blockers
Ca: Calcemia
P: Phosphatemia
SCr: Serum creatinin
SCysC: Serum cystatin C.
